# Supplementary material for: Heterogenization of Heteropolyacid with Metal-Based Alumina Supports for the Guaiacol Gas-Phase Hydrodeoxygenation
Source: Molecules. 2023 Feb 28;28(5):2245. doi: 10.3390/molecules28052245 (PMC10005010; doi:10.3390/molecules28052245)
Supplement: Supplementary file 1 [file molecules-28-02245-s001.zip › molecules-2251431-supplementary.pdf]

Heterogenization of Heteropolyacid with Metal-Based Alumina Supports for The Guaiacol Gas-Phase Hydrodeoxygenation

Structural Properties

The Figure S1 presents pattern of $H_3PW_{12}O_{40}$ sample and the COD data of $Cs_6ZnW_{12}O_{40}{\cdot}8H_2O$ for structure comparison purposes with $Cs_{2.5}$ salt.

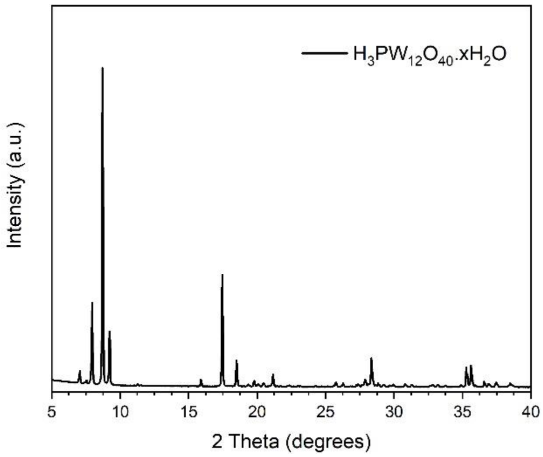

***Figure S1**: PXRD patterns of the starting heteropoly acid material $H_3PW_{12}O_{40}.xH_2O$.*

Optical Properties

Figure S2 show Infrared spectra of the starting HPW material.

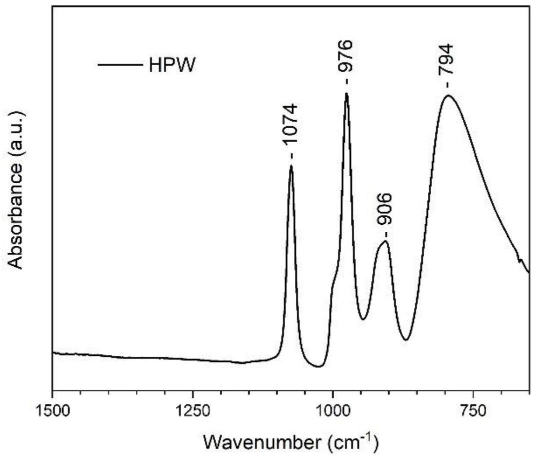

***Figure S2:** Infrared spectra of the starting heteropoly acid material acid material $H_3PW_{12}O_{40}.xH_2O$.*

XPS Results

Table S1: Experimental atomic ratios obtained from XPS experiments. Comparison with predicted values.

Sample	W/P		Cs/P		Pt/Al		Ni/Al		O/W		P/Al	
	Exp[a]	Th[b]	Exp[a]	Th[b]	Exp[a]	Th[b]	Exp[a]	Th[b]	Exp[a]	Th[b]	Exp[a]	Th[b]
**HPW**	6.9	12	-	-	-	-	-	-	4.0	3.3	-	-
**$Cs_{2.5}$ salt**	9.0	12	2.6	2.5	-	-	-	-	4.6	3.3	-	-
**Pt-Al_2O_3**	-	-	-	-	0.006	0.005	-	-		-	-	-
**HPW/Pt-Al_2O_3**	2.9	12	-	-	0.005	0.005	-	-	48.4	3.3	0.022	0.005
**Pt-Al_2O_3/$Cs_{2.5}$**	6.9	12	2.5	2.5	-	0.005	-	-	13.3	3.3	0.042	0.004
**Ni-Al_2O_3**	-	-	-	-	-	-	0.19	0.34		-	-	-
**HPW/Ni-Al_2O_3**	3.0	12	-	-	-	-	0.16	0.34	65.5	3.3	0.011	0.006
**Ni-Al_2O_3/$Cs_{2.5}$**	7.6	12	3.1	2.5	-	-	0.29	0.34	8.1	3.3	0.087	0.005

[a]Experimental atomic ratios obtained from XPS experiments; [b]predicted atomic ratios from synthesis conditions.

Acidity Properties

Figure S3 presents the NH_3-TPD profiles of the starting materials.

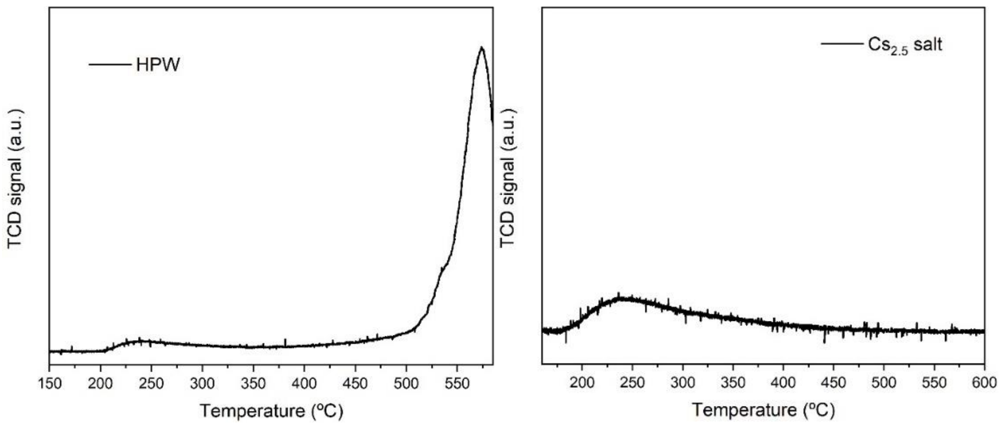

***Figure S3**: NH_3-TPD profiles of starting compounds: HPW (left); $Cs_{2.5}$ salt (right).*
